# Impact of Percutaneous Coronary Interventions (PCIs) on Health Outcomes from a Jordanian Patient’s Perspective

**DOI:** 10.3390/healthcare13131491

**Published:** 2025-06-23

**Authors:** Ahmad Hussein Al-Duhoun, Anees Adel Hjazeen, Maha Atout, Amjad Wasfi Fadeel Bani Salameh

**Affiliations:** 1 Faculty of Nursing, Mutah University, Al-Karak P.O. Box 61710, Jordan; 2 Royal Medical Services, Amman P.O. Box 855122, Jordan; aneesadel84@yahoo.com; 3 Nursing School, Philadelphia University, Jarash Road, Amman P.O. Box 19392, Jordan; 4 Emergency Department, Jordan University Hospital, Amman P.O. Box 13046, Jordan; amjadjoker8@gmail.com

**Keywords:** percutaneous coronary intervention, health outcome, Jordanian, patient’s perspective

## Abstract

**Background:** Patients diagnosed with coronary artery disease (CAD) have been seen to exhibit increases in health-related quality of life (HRQoL) following percutaneous coronary interventions (PCIs). This study thus aimed to assess the impact of PCI on health outcomes among Jordanian patients three months post-procedure. **Methods:** This prospective descriptive study evaluated health outcomes three months post-PCI among Jordanian patients who had been originally diagnosed with Chronic Coronary Syndrome (CCS) before being scheduled for PCIs. Quantitative data was collected using the updated version of the Coronary Revascularization Outcome Questionnaire (CROQ v2) across a non-probability sample, based on accessibility, of Jordanian patients who had received the procedure at any of several hospitals in Jordan. Multivariate analysis of covariance (MANCOVA) was employed to examine the mean scores of patient-reported outcomes following revascularization, while partial correlations were employed to investigate associations among patients’ age, weight, gender, and the reported results. **Results:** A total of 101 patients participated in the study, with a predominance of males (*n* = 85, 84.2%) relative to females (*n* = 16, 15.8%). The results indicated a statistically significant improvement across all measures assessed across these patients. Furthermore, the results demonstrated that males showed higher physical function, psychological functioning, and cognitive performance relative to females following coronary revascularization surgery. Nevertheless, the results also revealed varied levels of adverse effects following coronary revascularization, with the most commonly reported being discomfort around the groin or arm wound, followed by pain in the same areas. Conversely, the least significant concerns pertained to the emergence of bruises and similar issues in the groin or arm areas where the catheter was inserted. **Conclusions:** This study shows that PCI improves CAD patients’ quality of life over the initial three-month period post-procedure. Understanding the positive associations of this and the negative consequences that it entails may help healthcare practitioners better identify those patients likely to benefit or suffer from PCI, enabling more appropriate interventions. To understand how PCI affects HRQoL in CAD patients over time, more research based on rigorous study designs and validated metrics is required, however.

## 1. Background

Coronary artery disease (CAD) is a condition caused by a buildup of plaque or other narrowing of the coronary artery that supplies the heart with blood and nutrients; this affects the blood flow to the heart, potentially leading to a heart attack. CAD is the commonest among global heart conditions, and it is also known as ischemic heart disease (IHD) or coronary heart disease (CHD). CAD is the leading cause of global morbidity, mortality and a major contributors to disability worldwide [1]. Globally, the prevalence of CAD in 2019 increased to 523 million people across the world; while the most common cardiovascular condition is coronary (ischemic) heart disease, the number of Coronary Vascular Disease (CVD) deaths recently also increased to 19.7 million [2].

Countries in the Middle East tend to have a high prevalence rate of CVD; however, there is no accurate health data regarding the mortality and morbidity caused by these diseases in the region. Epidemiological studies in Middle Eastern populations are relatively rare with respect to the incidence of CAD, despite coronary heart disease being the leading cause of morbidity, mortality, and disability in the Middle East and North Africa [3]. Jordan is a relatively small country in the Middle East, with a mean area of 89,332 km^2^ and a population of 11.44 million [4]. CAD is becoming a major public health problem in the country due to considerable change in social, territorial, dietary, and lifestyle habits among the population in recent decades [5]. In Jordan, CVDs are thus a leading cause of mortality, with increasing overall mortality numbers and healthcare costs emerging over time [6].

Many methods are currently used to manage CAD, and these range from medical treatments to percutaneous coronary intervention (PCI) and coronary artery bypass grafting (CABG). Coronary revascularization references the restoration of perfusion, typically by surgical means, and vascular bypasses and angioplasty are the two primary means of revascularization currently in use. PCI has become a cornerstone in the management of CAD, offering a less invasive alternative to surgical revascularization while still significantly improving both symptoms and myocardial perfusion [7]. Globally, PCI has been associated with improved clinical outcomes, reduced symptoms, and enhanced quality of life [8].

PCI has thus become an accepted medical practice in Jordan. Casual coronary angioplasty was first used in Jordan in 1987, and since the turn of the century, PCI and Percutaneous Transluminal Coronary Angioplasty (PTCA) have become much more common in Jordan. Over twenty health facilities now offer these procedures for Jordanians. According to Alhaddad et al. [9], the First Jordanian PCI Registry was developed due to significant changes in patterns of CVDs and treatment modalities. The clinical assessment of PCI outcomes thus incorporates evaluation of clinical outcomes, yet the success of PCI must be measured not only by improvements in hard clinical outcomes, such as reductions in ischemic events, enhanced left ventricular function, and decreased hospital readmissions, but also by assessing patients’ symptomatic relief and any quality-of-life improvements. Using health outcomes to form clinical judgments of PCI is a well-established practice, and numerous studies have been conducted on the topic [10], as understanding patient perspectives on PCI is critical for optimizing care pathways. However, to the best of the researcher’s knowledge, specific research focusing on the impact of PCI on health outcomes from a Jordanian patient perspective is highly limited. This study therefore aims to assess the impact of percutaneous coronary intervention (PCI) on health outcomes from the perspective of Jordanian patients at a point of three months post-procedure. Additionally, it seeks to identify individual predictors that may affect health outcomes following PCI based not on clinical results but instead on quality of life, physical function, and psychological well-being.

## 2. Materials and Methods

### 2.1. Design and Sample

This study utilized a prospective descriptive design to evaluate health outcomes three months post-PCI from a Jordanian patient’s perspective. Quantitative data was collected using the modified version of the Coronary Revascularization Outcome Questionnaire (CROQ v2) [11].

A non-probability sampling technique based on accessibility was employed to recruit Jordanian patients who had undergone PCI at any one of a number of Jordanian hospitals offering this procedure. Participants who completed the pre-revascularization questionnaire were followed up three months after undergoing PCI to complete a post-revascularization questionnaire. Follow-up reminders were sent via WhatsApp, email, and other messaging platforms, based on each participant’s preferred method of communication, to encourage completion of the second part of the questionnaire. The main precipitating event for PCI in this study was Chronic Coronary Syndrome (CCS), with such patients primarily presenting with stable angina. Eligible participants were defined as adults aged 18 to 65 years who had been diagnosed with CCS based on clinical evaluations in accordance with the 2019 European Society of Cardiology (ESC) Guidelines for the Diagnosis and Management of Chronic Coronary Syndromes [12]. All patients underwent initial non-invasive testing, such as exercise stress testing or stress echocardiography, to evaluate myocardial ischemic status. Any decision to proceed with PCI was subsequently confirmed by diagnostic coronary angiography demonstrating significant coronary artery stenosis. Only patients with a confirmed diagnosis of CCS who were scheduled for elective PCI were thus included in the study.

The inclusion criteria thus focused on Jordanian patients aged 18 to 65 years who underwent PCI in one of three key Jordanian hospitals located in Amman, Jordan’s capital, and who were proficient in speaking, understanding, and writing Arabic. Exclusion criteria included non-Jordanian patients; those with additional cardiac conditions or physical disabilities; and individuals unable to speak, understand, and write Arabic.

The three Jordanian hospitals were as follows:

- Queen Alia Heart Institute: As part of the Jordanian Royal Medical Services (RMS), this institute specializes in cardiac care.
- Prince Hamza Hospital: This 436-bed general hospital was established in 2006 as part of the Jordanian Ministry of Health (MOH), and it offers a range of medical services.
- Jordan University Hospital: This teaching hospital, affiliated with the University of Jordan, provides comprehensive medical services, as well as serving as a center for medical education and research.

Demographic data collected from participants included age, gender, and weight. The ensuing analysis thus explored associations between these characteristics and health outcomes three months post-PCI.

The required sample size for the current study was determined based on effect-size (Cohen’s dz = 0.297) Cohen [13], with G-power software version (3.1.9.7) used to calculate effect size with a study power of 0.80 and a two-tailed alpha error of 0.05, which yielded a required sample size of 91 participants. However, given the prospective nature of the study, the sample size was increased to 160 to minimize the risk of incomplete responses and potential nonresponse bias.

Only data from participants who completed both the pre- and post-PCI questionnaires was included in the final analysis. The total number of participants who met the criteria and whose data was thus analyzed in the study was 101. Recruiting this larger sample was challenging due to the logistics of data collection, which required identifying patients scheduled for PCI and conducting follow-up assessments at three months post-procedure. This process contributed to participant attrition, representing a limitation of the study related to sample size.

### 2.2. Instruments

The CROQ, which was developed by Sara Schroter and Donna Lamping [14], has more recently been modified to CROQv2 [11]. For this study, the modified CROQv2 was thus used. This is a self-administered instrument with patient-reported outcome measures used to evaluate health outcomes and health-related quality of life before and after PCI. CROQv2 is currently the sole disease-specific instrument created explicitly to assess health outcomes prior to and following cardiac revascularization [11]. The questionnaires developed using it thus focused on symptom improvement, functional status, and quality-of-life metrics. CROQ has four parts: the CROQ-CABG and CROQ-PCI evaluate health outcome pre- and post-procedures. All four versions of CROQv2 contain a core set of 32 item; however, are scored across four scales: these are the symptoms scale (seven items), which provides an estimate of the impact of a range of symptoms; the physical functioning scale (eight items), which provides an estimate of the impact of the patient’s heart condition on their ability to perform usual physical daily activities; the psychological functioning scale (14 items), which provides an estimate of the psychological impact of the patient’s heart condition; and the cognitive functioning scale (three items), which provides an estimate of the effect of patient’s heart condition on their ability to perform everyday cognitive tasks.

CROQv2 was systematically translated into Arabic to ensure linguistic accuracy and cultural appropriateness. Initially, two Arabic-speaking researchers, each holding a Ph.D. in nursing, who are proficient in both English and Arabic, collaboratively translated the CROQ from English into Arabic, developing a consensus revised version. This agreed-upon Arabic translation was then provided to two additional Arabic-speaking researchers, who were blinded to the original English version. These researchers conducted back-translation of the reconciled Arabic version into English, and the resulting back-translated questionnaire was compared to the original English version to identify any inconsistencies or deviations in meaning, thus ensuring the translated version accurately reflects the original content.

### 2.3. Reliability Analysis

The revised coronary revascularization questionnaire (CROQv2) is a reliable and valid tool when used to collect patient outcomes before and after revascularization [11]. However, for this study, a pilot study of 10 patients was also completed to test the reliability coefficients of relevant items for internal consistency. The tool features several domains, including symptoms, physical function, psychosocial, and cognitive function, as well as noting adverse effects and satisfaction by means of the post-revascularization questionnaire. Cronbach’s alpha coefficients were generated for each domain, and these were at a satisfactory level (all domains had Cronbach’s alpha coefficients above 0.70), confirming the reliability or stability of the study tool.

### 2.4. Statistical Analysis

The categorical data was expressed in terms of frequency and percentage, while the scale data was presented using mean, SD, and range. A paired sample *t*-test with Bonferroni correction was applied to investigate improvements in scores in patients’ reported outcomes before and after revascularization. Multivariate analysis of covariance, MANCOVA, was used to investigate patients’ reported outcome mean scores (symptoms, physical, psychosocial, and cognitive function) after revascularization across their categorical demographics after controlling baseline measures as covariates. Partial correlations were used to explore the relationships between patient age, weight, gender, and reported outcomes after removing the effects of the control variable (baseline reported outcomes). Any *p*-values less than 0.05 were deemed statistically significant when data was analyzed using IBM SPSS (Version 27).

### 2.5. Ethical Considerations

Approval from the Institutional Review Board (IRB) at Mutah University in Al-Karak, Jordan, was sought, along with that of the IRBs of the selected hospitals. These were obtained before the study of Jordanian patients who had undergone PCI in the three selected hospitals. The study team collected data by approaching patients scheduled for PCIs. Each prospective participant received an invitation detailing the study’s objectives that highlighted the fact that participation was entirely voluntary and anonymous, and that participants could withdraw from the study at any time, without penalty, but that their information would remain strictly confidential and used solely for research purposes. Patients who consented to participate were also required to sign a consent form before completing the CROQv2 (Pre-PCI). A follow-up was conducted three months after their PCIs, with participants being asked to complete the CROQv2 (post-PCI). All collected data is securely stored in a locked drawer within the office of the primary researcher to be retained for a period of five years to ensure confidentiality and compliance with data-retention policies. Authorization to use the research instrument in this study was obtained by formally contacting the original developer, who offered explicit permission for such use.

## 3. Results

A total of 101 patients participated in this study. The vast majority of these were males (*n* = 85, 84.2%), with few female patients (*n* = 16, 15.8%). The study collected data from three major cardiac healthcare facilities in Jordan, though most patients (*n* = 45, 44.6%) were treated at Jordan University Hospital. The average age and body weight were found to be 56.97 (SD = 8.50) and 83.14 kg (SD = 13.66), respectively. Further details are given in Table 1.

Among the 101 participants, 10.9% (*n* = 11) had a history of hypertension (HTN), while 12.9% (*n* = 13) were diagnosed with diabetes mellitus (DM); 76.2% (*n* = 77) reported no comorbid conditions. With respect to smoking status, 39.6% (*n* = 40) of participants were current smokers, 33.7% (*n* = 34) were non-smokers, and 26.7% (*n* = 27) identified as former smokers.

### 3.1. The Effect of Coronary Revascularization on Patient Symptoms, and Physical, Psychosocial, and Cognitive Function

The results in Table 2 present the mean CROQ scores for the sample, as evaluated before and after coronary revascularization. There was a statistically significant improvement across all measured scales among PTCA patients, with patients reporting a substantial reduction in symptoms. The average score for these increased from 41.22 (SD = 23.36) before procedures to 68.71 (SD = 24.41) after procedures, yielding a large effect size (Cohen’s d = 0.992; *p* < 0.001). Additionally, physical function improved significantly, with the mean score increasing from 46.91 (SD = 30.69) to 66.52 (SD = 30.69), demonstrating a moderate effect size (Cohen’s d = 0.576; *p* < 0.001). Psychosocial function also showed a significant improvement from 56.84 (SD = 26.41) to 68.09 (SD = 23.38); however, this change yielded only a small effect size (Cohen’s d = 0.468; *p* < 0.001). Finally, cognitive function displayed notable enhancement, increasing from 67.79 (SD = 26.87) to 82.05 (SD = 23.22), with a moderate effect size (Cohen’s d = 0.502; *p* < 0.001).

### 3.2. Mean Differences in Coronary Revascularization Outcomes According to Patients’ Demographics

The MANCOVA test showed that gender had an impact on physical function scores (*F* = 13.376; *p* < 0.001), with males (*M* = 71.62; *SD* = 28.16) reporting higher physical function than females (*M* = 39.45; *SD* = 30.17). Similarly, males (*M* = 71.03; *SD* = 21.32) reported higher psychosocial function compared to females (*M* = 52.45; *SD* = 28.11; *p* = 0.043). Male patients also reported higher cognitive function (*M* = 84.63; *SD* = 20.29) as compared to females (*M* = 68.33; *SD* = 32.43; *p* = 0.045) after coronary revascularization procedures. However, hospital, patient age, and weight had no significant impact on patients’ coronary revascularization outcomes (Table 3).

### 3.3. Patients Demonstrating Adverse Effects Post-Revascularization

Table 4 illustrates varying degrees of adverse effects after coronary revascularization, with lower scores reflecting more serious adverse effects. Among the reported adverse events, the highest level of concern was related to related tenderness around groin or arm wound (*M* = 3.67; *SD* = 1.30), followed by pain in a groin or arm wound (*M* = 3.74; *SD* = 1.28). The least pronounced concern was the appearance of bruises (*M* = 4.26; *SD* = 1.13) and problems in the groin or arm where a catheter was inserted (*M* = 4.34; *SD* = 1.10). Patients’ satisfaction was also assessed post-revascularization as well. The respondents offered a satisfaction mean score of 71.38 out of 100, SD = 21.56, with a range from 11 to 100. This indicated an overall moderate satisfaction level after revascularization therapy.

## 4. Discussion

The aim of this study was to examine Jordanian patients’ health outcomes three months after PCI procedures. Additionally, it aimed to uncover individual PCI health outcome determinants. The findings of this study demonstrated statistically significant improvements across all health outcomes three months after PCI procedures from Jordanian patients’ perspectives, with outcomes including symptoms, physical function, psychosocial function, and cognitive function, with varying values for each category. This finding is congruent with previous studies [15,16]. Takousi [16] employed mixed methods to investigate Greek patients with coronary heart disease (CHD) with respect to their perspectives on health-related quality of life (HRQoL) during cardiac rehabilitation (CR). The findings in that study suggested that symptom scores, physical functioning, and psychosocial and cognitive functioning demonstrated increases over both a three-month period and a subsequent one-year period. Siriyotha, Pattanaprateep, Srimahachota, Sansanayudh, Thakkinstian and Limpijankit [15] similarly showed that, following PCI, the mean HRQoL of Thai patients improved significantly, with scores significantly increasing at both 6 and 12 months.

The findings of the current study also indicate that gender influences physical function scores, with males reporting higher physical function than females. Likewise, males reported superior psychosocial functioning in comparison to females, alongside superior cognitive performance relative to female patients following coronary revascularization operations. This result also aligns with prior studies [15,17], suggesting that males experience greater improvements in HRQoL following PCI. This is demonstrated by a reduced incidence of angina, enhanced physical functioning, and higher health-related quality of life in comparison to females. Conradie, et al. [18] similarly found that female patients’ self-reported quality of life was significantly inferior to that of men. Indeed, women consistently report inferior health-related quality of life, irrespective of the assessment method employed in comparable studies [19,20].

Several factors might contribute to males’ better outcomes after PCI. First, males often possess larger coronary arteries and have less diffuse disease in comparison to females [21]. Further, males usually tend to have CAD at an earlier age, which might contribute to a reduction in risk factors and comorbidities [21,22]. Females typically exhibit coronary artery disease at more advanced stages, leading to postponed detection and intervention [23]. Potentially, social and psychological issues also have a more significant impact on women, based on their reduced access to healthcare as compared to men [18,24].

The findings of the present study also indicate that hospital, patient age, and weight did not significantly influence the outcomes of coronary revascularization. This is contrary to previous research findings regarding the influence of body weight on alterations in HRQoL associated with PCI. The results of some studies have suggested that individuals who are overweight or obese frequently exhibit superior physical performance and overall quality-of-life scores in comparison to those with normal or lower BMIs [15,25,26]. This inconsistency in results may be due to shortcomings in research design. For example, the sample size may be inadequate to establish a significant correlation, or the convenience sampling strategy may potentially have impacted the external validity of the study. Consequently, performing a study utilizing a larger representative sample would enhance the capacity to determine any such possible relationship.

The findings of the current study also illustrate differing levels of unfavorable effects following coronary revascularization. The most significant adverse event reported was tenderness around the groin or arm wound, followed by pain in these areas. Conversely, the least significant concerns pertained to the emergence of bruises and issues in the groin or arm where a catheter was sited. These findings emphasize the significance of pain management and site care following PCI, leading to this study advocating for patients to be informed of all potential adverse effects, based on the idea that interventions such as pain management medication and cold treatment may alleviate such symptoms and enhance patient experience.

## 5. Limitations

This study is not without its limitations. The sample was neither truly large nor randomized. Further, although the study was conducted in three different hospitals, representing the three health sectors in Jordan, the findings remain difficult to generalize, as all three hospitals were located in Amman City. Amman is the capital city of Jordan, and, as such, any findings may not be generalizable to other locations in Jordan, particularly the northern and southern regions. Additional studies to fully explore these areas are thus required. Moreover, this study was conducted with a Jordanian population, who may possess distinct traits that could complicate the generalization of findings to other populations.

As the current study sample featured solely those patients who had received PCI, the examined group does not represent the entirety of patients with coronary artery disease. Moreover, despite attempts to mitigate biases, the inherent nature of the study may have engendered social desirability bias, as participants might have sought to answer the questions in a socially acceptable manner. There is also a lack of defined measures for health-related quality of life (HRQoL) or utility evaluations, based on the existence of various divergent measurements. This study utilized CROQ v2, limiting comparability with the results of other research using different measures.

A further limitation is that the study examined HRQoL among patients only three months post-PCI. This duration may be insufficient to assess long-term quality of life, highlighting the need for further studies to evaluate quality of life in the longer term (12 months post-procedure, for example).

## 6. Conclusions

The results of this study offer further evidence of the positive impact of PCI on quality of life among CAD patients over a three-month duration post-procedure. This study found that being male is linked to enhancements in HRQoL following PCI. Moreover, the results revealed that tenderness in the groin or arm wound was the main cause for concern among reported adverse events. Understanding the various correlations and unfavorable effects may enable healthcare professionals to identify patients who are likely to experience improved or worsened health-related quality-of-life outcomes following PCI, thus facilitating more appropriate interventions. Additional research utilizing robust study designs and established measurements is, however, essential to better elucidate the long-term effects of PCI on HRQoL alterations in individuals with CAD.

## Figures and Tables

**Table 1:** Socio-demographic characteristics of study sample (*n* = 101).

Variables	Category	Frequency	Percentage	Mean (SD)
Age in years				56.97 (8.50) Range 24–65
Weight in kg				83.14 (13.66) Range 60–140
Gender	Male Female	85 16	84.2 15.8	
Comorbidity	HTN DM No reported comorbidities	11 13 77	10.9 12.9 76.2	
Smoking status	Smoker Non-smoker X-Smoker	40 34 27	39.6 33.7 26.7	
Hospitals	Queen Alia Heart Institute (RMS) Jordan University Hospital Prince Hamzah Hospital (MOH)	29 45 27	28.7 44.6 26.7	

**Table 2:** Coronary revascularization outcomes before and after coronary revascularization.

Coronary Revascularization Outcomes	Before	After	Test Value	* *p*-Value	Effect Size	Cohen’s d
	Mean (SD)	Mean (SD)				
Symptoms	41.22 (23.36)	68.71 (24.41)	9.969	<0.001	0.992	Large effect
Physical function	46.91 (30.69)	66.52 (30.69)	5.788	<0.001	0.576	Moderate effect
Psychosocial function	56.84 (26.41)	68.09 (23.38)	4.704	<0.001	0.468	Small effect
Cognitive function	67.79 (26.87)	82.05 (23.22)	5.064	<0.001	0.502	Moderate effect

* High scores reflect high improvement. * Bonferroni correction *p* < 0.012 considered significant; 0.2 (small effect), 0.5 (moderate effect), and 0.8 (large effect) (Cohen, 1998 [13]).

**Table 3:** Mean differences in coronary revascularization outcomes by patient demographics.

Variables	Category	Coronary Revascularization Outcomes			
		Symptoms Mean (SD)	Physical Mean (SD)	Psychosocial Function Mean (SD)	Cognitive Mean (SD)
Gender	Male Female	69.27 (24.41) 65.73 (24.98)	71.62 (28.16) 39.45 (30.17)	71.03 (21.32) 52.46 (28.11)	84.63 (20.29) 68.33 (32.43)
	F (*p*-value)	0.007 (0.935)	13.376 (<0.001)	4.206 (0.043)	4.109 (0.045)
Hospitals	Queen Alia Heart Institute Jordan University Hospital Prince Hamzah Hospital	70.93 (22.44) 65.84 (25.24) 71.11 (25.39)	74.78 (27.47) 58.61 (31.26) 70.83 (30.86)	71.98 (17.28) 62.62 (26.36) 73.02 (22.61)	85.52 (16.34) 79.85 (26.23) 81.98 (24.52)
	F (*p*-value)	0.738 (0.481)	2.121 (0.126)	1.565 (0.214)	0.149 (0.862)
Age	R (*p*-value)	−0.132 (0.197)	−0.053 (0.608)	−0.184 (0.072)	−0.040 (0.698)
Weight	R (*p*-value)	−0.042 (0.681)	−0.055 (0.593)	−0.107 (0.298)	−0.091 (0.367)

Note: r, Pearson correlation; SD, standard deviation.

**Table 4:** Common adverse effects post-revascularization.

Adverse Effect	* Mean	SD
Tenderness around groin or arm wound	3.67	1.30
Pain in groin or arm wound	3.74	1.28
Bruising around groin wound, thigh, or arm wound	3.92	1.19
Numbness or tingling in groin area or around wound	4.14	1.18
Concern over the appearance of bruises	4.26	1.13
Problem in groin or arm where the catheter was inserted	4.34	1.10

* Lower score reflects a more significant adverse effect.

## Data Availability

The data presented in this study are available on request from the corresponding author.
